# Corrigendum to “Investigation of Hepatoprotective Activity of Induced Pluripotent Stem Cells in the Mouse Model of Liver Injury”

**DOI:** 10.1155/2022/9794832

**Published:** 2022-11-26

**Authors:** Chih-Hung Chiang, Ching-Chih Chang, Hui-Chun Huang, Yi-Jen Chen, Ping-Hsing Tsai, Shaw-Yeu Jeng, Shuen-Iu Hung, Jung-Hung Hsieh, Hsu-Shan Huang, Shih-Hwa Chiou, Fa-Yauh Lee, Shou-Dong Lee

**Affiliations:** ^1^Institute of Pharmacology, National Yang-Ming University, No. 155, Section 2, Linong Street, Taipei 11221, Taiwan; ^2^Division of Urology, Department of Surgery, Taipei Veterans General Hospital and Su-Ao & Yuan-Shan Branch, No. 201, Section 2, Shih-Pai Road, Taipei 11217, Taiwan; ^3^School of Medicine, National Yang-Ming University, No. 155, Section 2, Linong Street, Taipei 11221, Taiwan; ^4^Division of General Medicine, Taipei Veterans General Hospital, No. 201, Section 2, Shih-Pai Road, Taipei 11217, Taiwan; ^5^Division of Gastroenterology, Taipei Veterans General Hospital, No. 201, Section 2, Shih-Pai Road, Taipei 11217, Taiwan; ^6^Department of Obstetrics and Gynecology, Taipei Veterans General Hospital, No. 201, Section 2, Shih-Pai Road, Taipei 11217, Taiwan; ^7^Graduate Institute of Pharmacy, National Defense Medical Center, No. 161, Section 6, Minquan E. Road, Taipei 11490, Taiwan; ^8^Department of Medical Research & Education, Taipei Veterans General Hospital, No. 201, Section 2, Shih-Pai Road, Taipei 11217, Taiwan

In the article titled “Investigation of Hepatoprotective Activity of Induced Pluripotent Stem Cells in the Mouse Model of Liver Injury” [1], a duplication within Figure 1(b) was mistakenly introduced. The authors have stated that this was due to an error when preparing the files for publication and have now repeated the experiment to validate the accuracy and reproducibility of their study.

To take into account the continuity of the hepatocyte differentiation from iPSCs to mature iPSC-derived hepatocytes, the authors have provided new images for Figures 1(a) and 1(b). Additionally, due to the poor resolution of Figure 2 in [1], the authors have provided a revised figure. New Figures 1 and 2 are shown below.

## Figures and Tables

**Figure 1:**
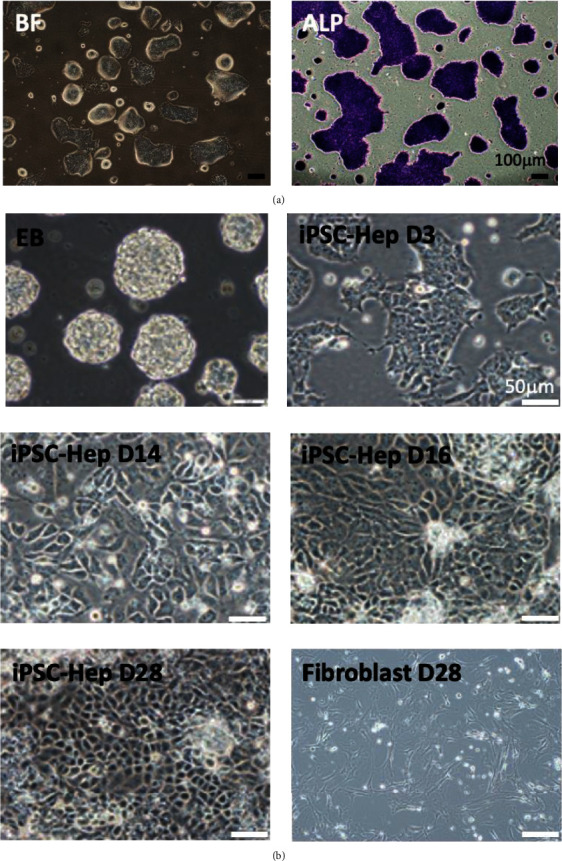
In vitro differentiation of iPSCs into iPSC-Heps. iPSCs were seeded at 2 × 10^4^ cells/cm^2^, maintained in Dulbecco's modified Eagle's medium. (a) Left: morphology of iPSC colonies. Right: iPSC colonies were positive for alkaline phosphate stain (purple). (b) The hepatogenic differentiation was induced by a 2-step procedure as described in Section 2. Morphology of undifferentiated and differentiated iPSCs was evaluated at different days after hepatogenic differentiation.

**Figure 2:**
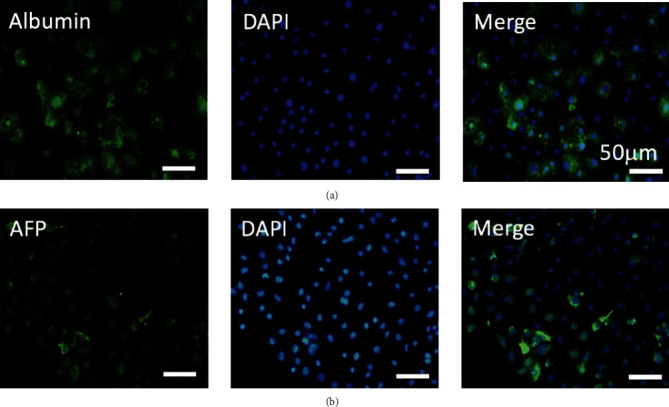
Immunofluorescence staining for several hepatocyte-specific markers in iPSC-Heps. Immunostaining imaging (800x) results showed that several hepatocyte-specific markers were detected by using (a) anti-AFP antibody and (b) anti-albumin antibody in iPSC-Heps. Blue signal indicated DAPI-positive cells.
